# Use of a mobile app and educational website to promote metabolic control in people with type 2 diabetes: study protocol for a multicenter clinical trial

**DOI:** 10.3389/frhs.2025.1624780

**Published:** 2025-11-17

**Authors:** Lubia Velázquez López, Gabriela Ortíz Ortíz, Miguel Klünder Klünder, Jenny Vilchis Gil, Ignacio Pineda del Aguila, Oswaldo Sinoe Medina Gómez, Jorge Escobedo de la Peña, “Entiendo mi diabetes” Investigators Group

**Affiliations:** 1 Clinical Epidemiology Research Unit, “Dr. Carlos Mac Gregor Sánchez Navarro” Hospital, Mexican Social Security Institute, Mexico City, Mexico; 2 Epidemiological Research Unit in Endocrinology and Nutrition, Hospital Infantil de México Federico Gómez, CDMX, Mexico

**Keywords:** type 2 diabetes, glycemic control, mobile health, smartphone app, medical education, online resource

## Abstract

**Background:**

Diabetes education is key to achieving metabolic control and promoting healthy behaviors in people with type 2 diabetes. Mobile health (mHealth) tools have been shown to be an important tool for monitoring, disease care and lifestyle improvement in people with type 2 diabetes (T2D). In less developed countries, the effect of educational intervention through mHealth is still inconclusive. The objective of this study is to evaluate the effect of an intervention on metabolic control in people with T2D with mHealth intervention and educational website called “I understand my diabetes”.

**Methods:**

The study is designed as a 12-month randomized controlled trial with three parallel arms: (1) Web-based education, (2) Web-based education + mobile app (diet and exercise plan), (3) Usual care. The total study duration is 12 months with data collection at baseline, 3, 6, 9 and 12 months. We will enroll 408 Mexican adults with T2D, randomized equally across arms. Measurements at baseline, 3, 6, 9, and 12 months include A1c (primary outcome), lipid profile, anthropometry, body composition, quality of life, lifestyle, and physical activity.

**Discussion:**

The trial will evaluate whether reinforcing diabetes education with an app and educational website improves metabolic outcomes in people with type 2 diabetes. Findings may be relevant for implementation in public institutions with high demand of people with type 2 diabetes who have not presented severe complications of the disease. Integration of mHealth into routine care could enhance self-management and disease control in people with type 2 diabetes.

**Clinical Trial Registration: Trial registration number:** Clinical Trials.gov**. Registry** (NCT0627857I). The protocol number**:** Effect of Education with Mobile App on metabolic control in Patients With type 2 Diabetes. The registration number R-2018-785-100. Instituto Mexicano del Seguro Social. Mexico. Registration date: February 22, 2024.

## Background

Type 2 diabetes (T2D) is a disease with a significant global impact, according to the International Diabetes Federation (IDF). By 2021, an estimated 536.6 million people (12.2%) were living with T2D, with projections reaching 783.2 million by 2045 (1). This condition is among the leading causes of mortality and disability in Mexico and worldwide (2, 3). The 2022 National Health and Nutrition Survey conducted in Mexico reported a diabetes prevalence of 18.3%, (5.6% newly diagnosed), which is higher than the 16.8% reported in 2018 (4).

The treatment of T2D should be considered by health professionals to promote the adoption of a healthy lifestyle that includes a balanced diet, regular physical activity, and motivation for effective self-care (5, 6).

In this sense, diabetes education should aim to provide patients with the knowledge required for effective self-management of T2D. This includes fostering the adoption of positive health behaviors, encouraging adherence to care recommendations provided by healthcare professionals, and promoting a healthy lifestyle. It has been reported that an adequate lifestyle can prevent up to 37% of macrovascular complications and 24.7% of microvascular complications in people with T2D (7).

The use of technology in the care and treatment of T2D has grown globally, particularly in high-income countries since the inclusion of tools for insulin calculating and tracking, as well as glucose monitoring, and graphing glucose levels (8, 9). In addition, mHealth technologies have been increasingly used to support diabetes education and self-management through mobile devices (10). There is also evidence supporting the effectiveness of mHealth for weight loss among people with T2D (11).

Moreover, m Health has shown potential in helping individuales meet therapeutic goals, including glycemic control through reductions in A1c levels and improvements in other cardiometabolic risk indicators, such as waist circumference (WC). It also contributes to more efficient clinical care of people with diabetes (12).

mHealth has been associated with improvements in self-management, medication adherence, communication with healthcare providers, and the adoption of healthy behaviors (13). However, the scope and effectiveness of its various applications remain under discussion—particularly regarding its use in glucose monitoring, diabetes education, lifestyle interventions (whether autonomous or guided by professionals), and the duration of follow-up strategies (14).

Current mHealth strategies commonly target glucose monitoring, dietary management and insulin dose adjustments, medication adherence, and physical activity. Nevertheless, the number of studies assessing their applicability in routine clinical care for adults with diabetes remains limited (12).

Furthermore, there is a lack of information about comprehensive strategies in developing countries that integrate monitoring, diabetes education, promoting healthy lifestyle habits, and self-care aimed at meeting therapeutic goals in developing countries (15). There is also limited evidence regarding validation of mHealth content by healthcare professionals before implementation. Previous studies have reported the feasibility, acceptability, and usefulness of providing diabetes education for patients through an educational website in both in-clinic and remote formats (16, 17).

In Mexico, 68% of people with T2D have poor glycemic control and limited adherence to non-pharmacological treatment. This is associated with a high prevalence of obesity, dyslipidemia, and arterial hypertension. These risk factors can affect the quality of life and increase costs for public health systems (18).

Our previous research has shown the positive effects of implementing an educational website in a clinical setting, used prior to medical and nutritional consultations. This intervention not only led to sustained improvements in A1c levels over 12 months but also enhanced diabetes-related knowledge and healthy lifestyle adoption among people with T2D (19–21).

The objective of the present study is to evaluate the effect of a digital intervention “I Understand My Diabetes” on metabolic control in people with T2D. The intervention includes a mHealth component and an educational website, designed and adapted to the needs of patients attending the health institute that serves the largest population of people with diabetes in Mexico. The intervention follows international clinical trial guidelines and recommendations (https://www.ctti-clinicaltrials.org).

## Methods/design

### Objectives

The primary objective of this clinical trial is to assess changes in A1c when comparing web-only, web + app, and usual care.

The secondary objectives are to evaluate the effect of the intervention on:

1. Lipid profile [total cholesterol, LDL-c [low-density lipoprotein cholesterol], HDL-c [high-density lipoprotein cholesterol], and triglycerides].
2. Body composition and anthropometric measurements (body fat percentage, body fat mass, weight, BMI [body mass index], and waist circumference).
3. Quality of life (Diabetes 39, validated in the Mexican population).
4. Lifestyle (IMEVID, validated for diabetes population in Mexico).
5. Level of physical activity [IPAQ (International Physical Activity Questionnaire)].

### Design and setting

A randomized controlled trial with three parallel groups, will be conducted across seven family medicine clinics of the Mexican Social Security Institute in Mexico City. The duration of intervention and follow-up is 12 months, with evaluations at baseline, 3, 6, 9 and 12 months. This study complies with international ethical standards and was approved by the National Research and Ethics Commission of the Mexican Social Security Institute, under the registration number R-2018-785-100. The clinical trial considers the effect of the mobile app + website vs. the website alone and a control group. It assumes a minimum clinically important difference (MCID) of 0.5% in HbA1c at 12 months. Based on the findings reported by Duke et al. (Cochrane 2009; ref. 23), who reported modest mean reductions in HbA1c with individual education (−0.3% in participants with a baseline HbA1c > 8%), a reduction of 0.5% was considered; a standard deviation (SD) of 1.3% was assumed based on reports from primary care cohorts and registry data, where the SD of HbA1c typically ranges from 1.1% to 1.3%. Using the formula to compare two means with *α* = 0.05 (two-tailed) and 80% power: *n* per group = 2 × (Z1 − *α*/2 + Z1 − *β*)2 × *σ*2/*Δ*2 With Z1 − 0.025 = 1.96, Z0.80 = 0.84, *σ* = 1.3, and *Δ* = 0.5, the required sample size was 106 participants per group. Taking into account a 20% loss, this resulted in 133 per group, and for greater conservatism, 136 participants per group were recruited (22).

### Selection criteria

The study will involve adults who have been previously diagnosed with T2D by their treating physician, with A1c > 7% and < 13%, with and without pharmacological treatment for diabetes (hypoglycemic agents or insulin). Participants >65 years old with ≤10 years of diabetes diagnosis

**Figure 1:**
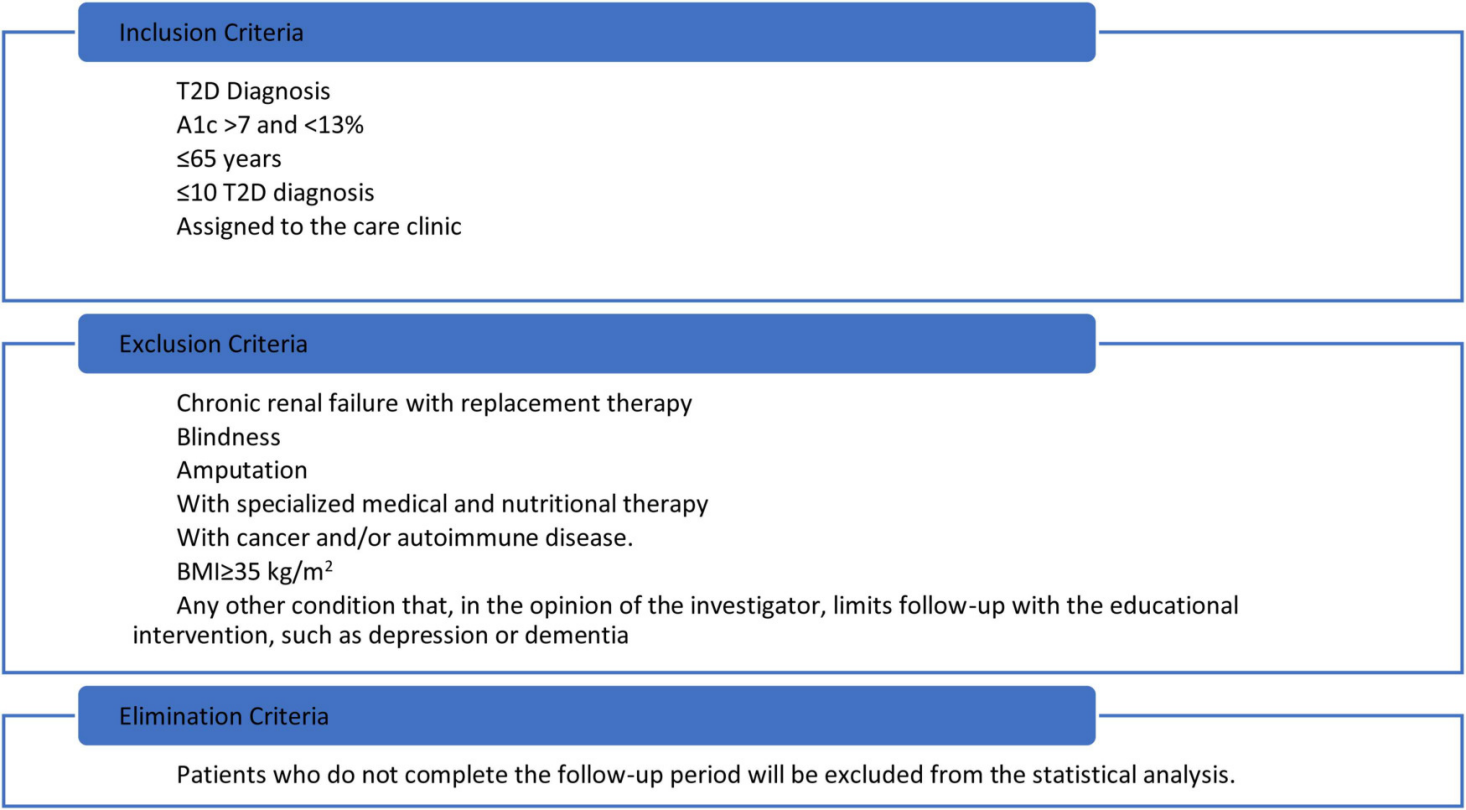
Selection criteria.

Participants with severe complications of the disease (chronic renal failure, blindness, or amputation), requiring specialized medical and nutritional care will be excluded as well as people with morbid obesity (BMI ≥ 35 kg/m^2^). Selection criteria for the study are described in Figure 1.

### Protocol management

The objectives and activities of the project will be explained to the authorities and permission will be requested to conduct the study at the selected clinics. The participating researchers will provide information about the study to the patients, answer their questions and, once people decide to participate voluntarily, ask for informed consent.

### Sociodemographic and clinical measurements

Information on sociodemographic data, pathological history, and clinical data (pharmacological treatment and current comorbidity) will be collected by the clinical staff. Blood pressure will be measured twice with a mercury sphygmomanometer with an interval of 5 min between measurements. Afterwards, the patient will remain seated for more than 5 min; the blood pressure value will be the average of the two measurements.

### Biochemical measurements

After a 12 h fasting period, venous blood samples will be obtained from participants. A1c levels will be measured using high-performance liquid chromatography. Serum levels of fasting glucose, total cholesterol, LDL-c, HDL-c, and triglycerides will be analyzed using automated photometry. They will be measured using the automated photometry method [Roche Cobas 800 c701). Samples, properly stored at all centers, will be sent in special containers to be processed at a certified central laboratory.

### Anthropometry and body composition measurements

Anthropometry will be recorded by two previously trained nutritionists and standardized according to the method proposed by Habitch and the specifications described by Lohman et al. (23, 24) Weight (kg) and height (cm) will be obtained for the calculation of BMI in an Inbody® device (model BPMO40S12F07). Additionally, WC will be measured at the midpoint between the lower rib and the upper edge of the superior iliac crest on the right side. Hip circumference (HC) will be measured at the greater diameter of the trochanters. The measurements will be recorded three times and the mean value of the second and third measurements will be used for analysis.

Body composition will be assessed using bioelectrical impedance analysis (BIA). At the UMF 20 clinic, measurements will be obtained with the InBody 120 device (InBody Co.), while at the remaining sites (UMF 1, 4, 7, 9, and 10), an Omron body composition monitor (Omron Healthcare) will be used.

All measurements will be performed under standardized conditions. Participants will be instructed to fast for at least 8 h, refrain from alcohol, caffeine, and strenuous exercise for 24 h prior to the assessment, and empty their bladder immediately before the test. Measurements will be taken with participants in light clothing, barefoot, and standing upright, following the manufacturer's operating guidelines for each device. The InBody 120 applies a multifrequency electrical current through hand and foot electrodes, while the Omron devices use a single-frequency current with footplate electrodes. Both methods estimate body composition using proprietary algorithms. The following variables will be recorded: body weight (kg), body mass index (BMI), body fat percentage (%), fat mass (kg), skeletal muscle mass (kg), and total body water (liters) will also be documented. To ensure consistency and reproducibility, all measurements will be conducted by trained personnel, using the same device for each participant at baseline and follow-up visits.

### Dietary measurements

A 24 h dietary recall will provide an overview of the diabetes participants' usual diet, from which the nutrition professional will evaluate the calories and macronutrients typically consumed, food quality, tastes, and preferences. The dietary measurement will be collected by the nutrition professional at each patient visit.

### Physical activity measurements

The IPAQ will be used to measure the level of physical activity. This questionnaire consists of 7 items that indicate the time of physical activities performed in the last 7 days (hours, minutes, and days per week). The physical activity will be classified after considering the METs (unit of measurement of the test) of the activity; then, physical activity will be classified in three categories: low, moderate, and high (25).

### Quality of life measurements

Quality of life will be measured using the Diabetes-39 questionnaire, which has been validated in the Mexican population. The questionnaire contains 39 items distributed in five sections: Energy-Mobility, Diabetes Control, Anxiety Concern, Social Burden, and Sexual Functioning. It is scored on a scale from 1 to 7, (1 for not at all affected and 7 for extremely affected in quality of life). The score of each section is transformed to a scale from 0 to 100 using a formula for linear conversion (26).

### Lifestyle measurements

The IMEVID instrument (Instrument for measuring lifestyle in people with diabetes) will be applied. It consists of 25 closed questions grouped into 7 dimensions: Nutrition, Physical Activity, Tobacco Consumption, Alcohol Consumption, Information on Diabetes, Emotional Management, and Compliance with Treatment. Each item presents three response options with scores of 0, 2, and 4, where 4 corresponds to the maximum desirable value in each response, for a total score from 0 to 100. (27) The IPAQ Instruments, Diabetes-39 questionnaire, and IMEVID instrument are validated instruments in Spanish.

### Clinical management

**Figure 2:**
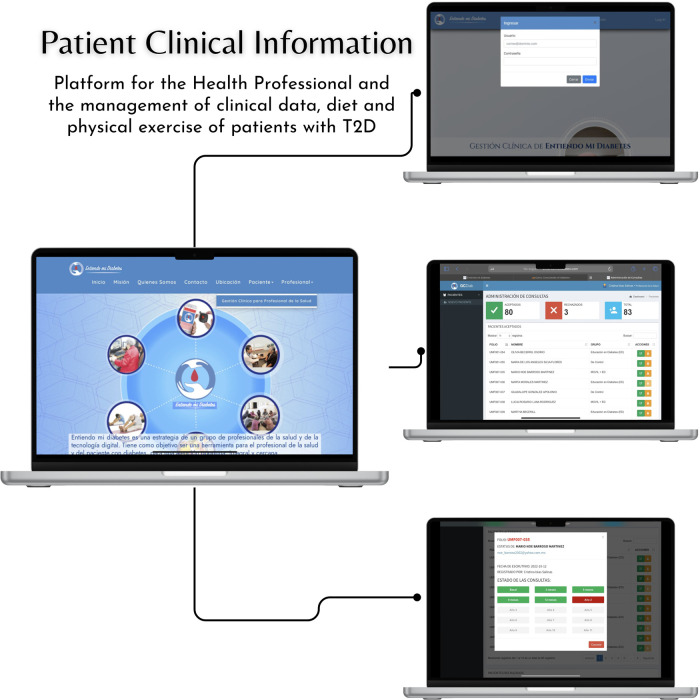
Patient clinical information.

The health professionals of each clinic (physician and/or nutritionist) will have credentials to access the clinical management platform “I Understand my diabetes” to capture sociodemographic, comorbidity, clinical, biochemical, anthropometric, body composition, lifestyle, quality of life, diet, and physical exercise data. From these data, the system will provide a diet and a physical exercise plan previously designed by physicians and nutrition researchers participating in the platform, considering the clinical data recorded by the health professionals. This clinical management system of the health professional can be seen in Figure 2.

All participants will receive a personalized dietary plan, designed by dietitians, based on their current weight and comorbidities. The plan will consist of 15%–20% protein, 50%–55% carbohydrates, and 25%–30% fat, with saturated fat accounting for less than 7% of total calories. The menus will range from 1,200–2,600 calories, tailored to address common comorbidities in individuals with diabetes, such as dyslipidemia, hypertension, and kidney disease, as well as personal preferences, dietary habits, and economic considerations.

### Randomization

**Figure 3:**
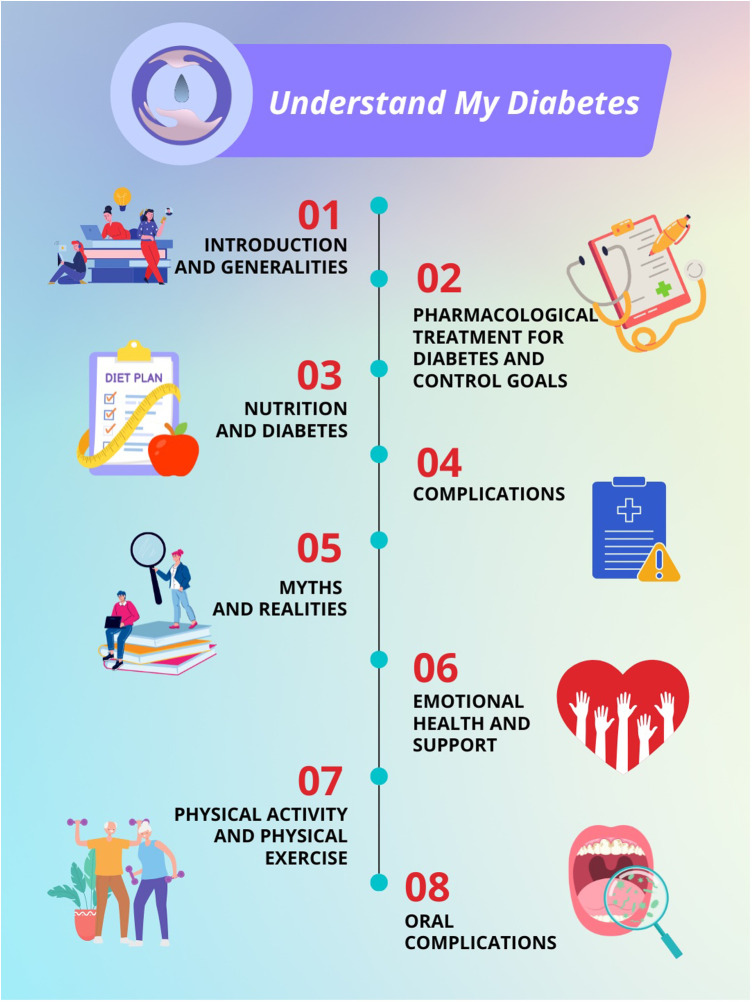
Educational website program.

**Figure 4:**
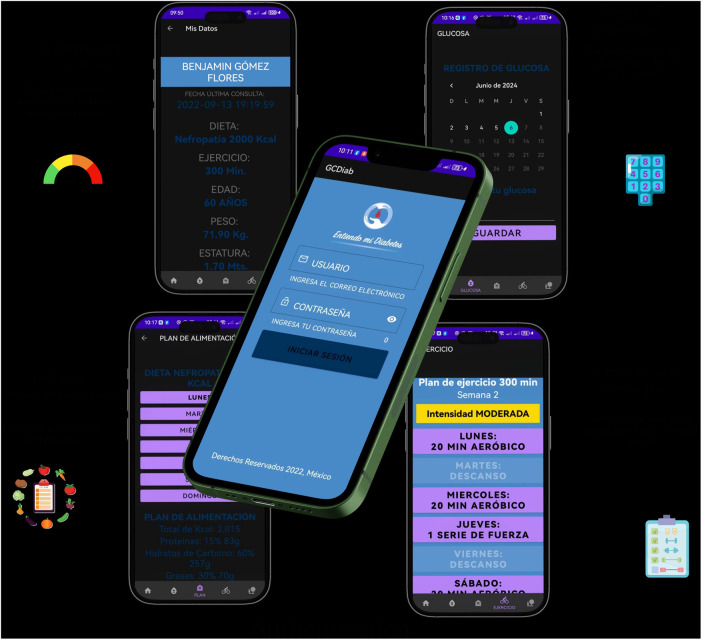
Mobile app.

Randomization of patients for the clinical trial will be performed using the National Cancer Institute's online statistical software, available at: https://ctrandomization.cancer.gov/tool/. Participants from each of the six clinics will be randomly assigned to one of the following three groups:

1. *Group with educational website program (*Figure 3*)*
2. *Group with mobile app* *+* *educational website program (*Figure 4*)*
3. *Group with usual care*

### Group with educational website program

Participants will receive the educational intervention with the website called “I Understand my diabetes”, designed by a group of clinical experts in diabetes, including physicians, nutritionists, diabetes educators, and epidemiologists. This educational site was previously validated through expert consensus and usability testing in individuals with type 2 diabetes (17, 28). The content, images, videos, and exposure time were previously validated through expert consensus, along with the usability, comprehensibility, and perceived usefulness among individuals with diabetes from the clinics where the intervention will be implemented. The educational course is available on the Moodle platform at https://entiendomidiabetes.org/.

The intervention includes ten interactive modules on topics such as diabetes education, pharmacological treatment, glycemic control indicators, nutrition, myths and facts about diabetes, complications, depression, and family support. Each module integrates visual aids, concise text, and videos to facilitate comprehension. Interactive activities within each module are designed to reinforce learning and promote active self-management of type 2 diabetes (T2D).

Participants will review one module per week, completing all modules in an organized manner over a two-month period. Thereafter, unrestricted access to the modules will remain available, allowing participants to revisit those of preference during the study follow-up period. At each in-person visit, emphasis will be placed on the modules reviewed and any questions regarding their content will be addressed. In addition, participants will receive a dietary plan tailored to their current weight and comorbidities, along with prototype menus to support dietary adherence. Upon completion of all modules, participants will be awarded a certificate of completion.

### Group with mobile app + educational website program

Participants assigned to this group will have access to the mobile app, where they can view their personalized diet and exercise plan, monitor their glucose and weight, and receive reminders to take the medications prescribed by their doctor. This group will have access to the website, and the aim is to improve adherence to drug treatment and lifestyle changes. The app displays the prescribed caloric intake and classifies foods using a color-coding system based on food group and glycemic index. In addition, participants will be able to make food exchanges that will allow them to vary their diet. They will also be able to see the evolution of their BMI and track their glucose and A1c levels as recorded by their treating physician. The app includes tools for physical activity, such as exercise routines and timers, as well as reminders for medical appointments. It records visits to the meal plan and tracks the times set aside for physical activity. The app was developed using a responsive architecture that enables real-time synchronization between healthcare professional management and the app. It incorporates interactive elements and full keyboard navigation. The follow-up system provides quantifiable metrics of therapeutic adherence through time-stamped records of medication intake, self-monitoring of blood glucose, nutritional tracking based on food exchanges, and physical activity. The app is currently undergoing usability testing, as shown in Figure 4.

### Group with usual care

Participants in the control group will receive general nutritional recommendations for following a healthy diet, as well as general measures for diabetes care. General physical activity recommendations will follow national diabetes care guidelines. In order to minimize follow-up losses, a communication link will be established with each participant's primary care provider, who will encourage continued participation. Appointments will be scheduled flexibly, and participants will receive reminders through the mobile app and follow-up calls.

### Follow-up and procedures

**Figure 5:**
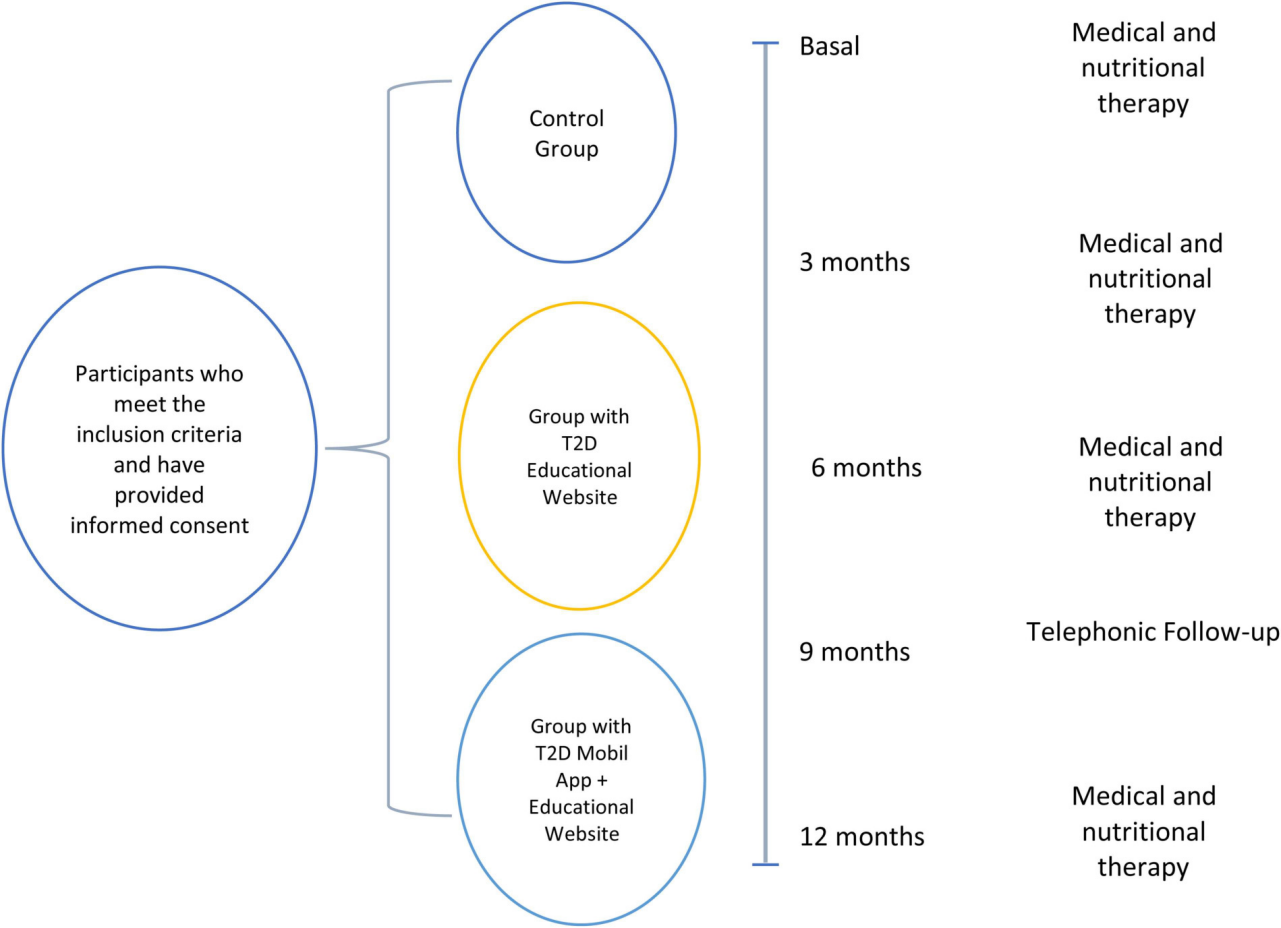
Follow-up and procedures.

The study will last 12 months. Participants will attend a face-to-face consultation every 3 months, as shown in Figure 5.

**Primary measurements**: A1c (Basal and 12 months).

**Secondary measurements**: Lipid profile. Anthropometry and body composition. Lifestyle. Quality of Life. Physical Activity Level. (Basal and 12 months). Medical and Nutritional Therapy. In the baseline phase, participants will receive a complete plan that will include dietary and physical exercise recommendations, together with the collection of sociodemographic, clinical and biochemical data. Blood pressure will be taken, and relevant questionnaires will be administered. At 12 months, biochemical and anthropometric data will be reevaluated, and appropriate instruments will be applied. Follow-up visits will be scheduled at 3 and 6 months to reinforce medical and nutritional interventions.

At baseline, a comprehensive assessment will be conducted, including dietary and physical activity recommendations, collection of sociodemographic, clinical and biochemical data, blood pressure measurements and administration of standardized questionnaires. These evaluations will be repeated at 6 and 12 months. Additional follow-up visits at months 3 and 6 will reinforce interventions. Visit time points and assessments are summarized in Table 1.

**Table 1:** Schedule of assessments.

Measurements during the intervention		Months			
	Basal	3	6	9	12
Medical examination to obtain sociodemographic and clinical data, measurement of systolic and diastolic blood pressure	X				
Biochemical data in blood samples (A1c, glucose, serum creatinine, total cholesterol, triglycerides, LDL-c and HDL-c).	X				X
Measurements of body weight, height, waist circumference, fat percentage, fat mass and lean mass	X	X		X	X
Medical and nutritional therapy, 24 h food reminder record.	X	X		X	X
Lifestyle (IMEVID), Quality of Life (Diabetes 39) Physical Activity Level (IPAQ)	X		X		X
Creation of users to the educational website and/or mobile App	X^a^				
Telephone call to reinforce the intervention group			X		

a For groups with educational website and mobile app + educational website.

**Figure 6:**
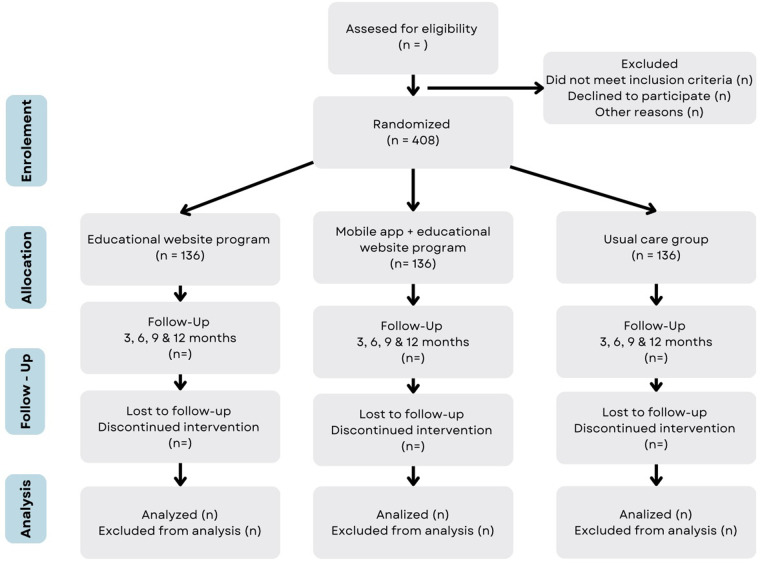
Flow diagram of the study. The expected flow of patients with diabetes in the different phases of the study.

The inclusion and follow-up of participants in the study is shown in Figure 6. The study follows the Standard Protocol Elements for Randomized Clinical Trials guidelines.

### Results of the study

Primary objective. To evaluate the effect on metabolic control through the measurement of A1c levels and fasting glucose before and after 12 months of intervention. The measurement will be considered a continuous variable to identify achievement in metabolic control from A1c (≤7%). Secondary objectives. To assess changes in lipid profile (total cholesterol, LDL-c, HDL-c, and triglycerides), and body composition (weight, BMI, fat percentage, fat mass, and waist circumference. After the intervention, it will be possible to compare the clinically significant results of the metabolic variables in control targets: HbA1c % (≤7%), fasting glucose mg/dl (≤130), cholesterol mg/dl (≤200), LDL-cholesterol mg/dl (≤100), HDL-cholesterol mg/dl (≥40 men and ≥50 women) and triglycerides mg/dl (≤150).

The assessment of lifestyle through IMEVID, validated in the population with diabetes in Mexico, will be treated as a categorical variable, ranging from adequate and regular to inadequate lifestyle. Quality of life will be assessed through Diabetes-39, which includes different components of quality of life and will be analyzed as a categorical variable.

### Statistical analysis

Descriptive statistics will be used with measures of central tendency and dispersion for quantitative variables that have a parametric distribution; variables with free distribution will be reported in median and interquartile range and will be presented in summary measurement tables. One-way ANOVA or Kruskal Wallis test will be used to compare the effect on clinical, anthropometric, and biochemical variables between groups. An X^2^ test will be used to evaluate the effect of the intervention between groups for metabolic control goals. An intention-to-treat analysis will be performed for the three groups in the clinical trial. In addition, a per-protocol analysis will be performed on the outcome variables according to compliance with the interventions. To this end, the level of compliance with the educational modules will be measured, as well as the use of the mobile application. A repeated measures ANOVA test will also be performed to analyze the effect of the intervention in each of the groups. A logistic regression model will be used to identify the effect of the intervention on the primary target variable, A1c, as a dichotomous variable in glycemic control and uncontrol. It will be adjusted for other variables, such as intervention group, years of diagnosis, physical exercise, schooling, marital status, and occupation.

## Discussion

This study evaluates the impact of the “I Understand My Diabetes” platform, a digital educational tool combining professional care with a self-paced learning model, on metabolic control among individuals with T2D.

Previous research by our team demonstrated a significant HbA1c reduction (–0.73%) after a 21-month website-based intervention in Mexico (18). Unlike the prior study, this intervention allows patients to access content asynchronously, enhancing accessibility and scalability.

Educational website content was validated by clinical experts and usability-tested in the target population.

Some of the limitations commonly reported in mHealth studies include the lack of content validation by healthcare professionals, as well as low acceptance and perceived usefulness by end users (13). Thus, the study performed content validation of the educational website through expert consensus (28) and measured the usability of the web content among end users (17).

It has been reported that technology-based interventions implemented in patient care clinics, with reinforcement by health professionals, improve knowledge about the disease and promote healthier lifestyles (20, 21).

Systematic reviews have documented the use of mobile apps for various purposes, ranging from collecting self-care data to evaluating healthcare professionals or patients (14). However, adherence to mHealth-supported strategies for monitoring, diet, physical exercise, and professional reinforcement tends to be higher in the short term but decreases over time (29). Therefore, improving usability may enhance adherence to mHealth interventions. The use of mHealth has been shown to improve lifestyle and reduce HbA1c in the short term, with smaller effects observed in the long term (12).

This study aims to evaluate the impact of delivering diabetes education via a website and mobile app that provide diet and physical exercise plans, while also linking with the health professionals' records, to provide more comprehensive information to the users.

The objectives of mobile app interventions vary widely, ranging from insulin dose control to improving diabetes knowledge and glucose monitoring through strategies such as text messages, health diaries, and virtual counseling (15). Although mHealth has been shown to complement diabetes care, particularly in developed countries, these tools should be regulated, designed by diabetes clinical experts, validated with clear objectives, tailored to users' needs, rendered easy to use, and focused on desired outcomes (30).

In Mexico, Internet and technology use in healthcare is increasing, both for care management and use by participants at home. However, socioeconomic, cultural, political, and economic barriers remain, alongside limited investment, low digital literacy among health professionals, and challenges implementing technology for diabetes care and prevention. It is necessary to design strategies that integrate digital tools into diabetes care, facilitating communication with physicians and empowering patient self-management.

The Mexican health system is divided into public and private sectors; the Mexican Social Security Institute (IMSS), part of the public sector, serves the largest proportion of the population, approximately 51.0% of those entitled in 2020 (31). By 2023, IMSS is projected to provide nearly 3.5 million consultations in primary care clinics to people living with diabetes (32).

Therefore, there is a significant opportunity to consolidate health promotion and secondary prevention strategies mediated by digital health and led by health professionals. This study seeks to evaluate the effectiveness of providing asynchronous diabetes education through a website with monitoring and promotion of a healthy lifestyle, using mHealth as a tool to improve metabolic control and healthcare outcomes.

### Data management

The data generated in the study will be stored in both physical repositories and digital media, with the responsible personnel verifying the capture date and time of each record. Data entry will utilize computer systems that ensure verification and readability. Information will be stored on digital media structured through an information architecture designed specifically for the study's purposes.

Data recording, retrieval, updating, and deletion will be performed securely, maintaining a historical record. Access to the registry and data retrieval will be controlled through access levels, permissions, and digital credential verification. System access will require user authentication.

### Data privacy and security

Before registering study participants in the mobile application, which handles sensitive data, a privacy notice will be displayed specifying the data that will be collected, how it will be used during the study, and the security measures in place to protect this information.

The notice will describe the sociodemographic, laboratory, clinical, and contact data required, clarifying that it will only be used by the Mexican Social Security Institute. Participants will also be informed of their rights regarding their data and provided contact information for any questions related to data use and security.

## “Entiendo mi diabetes” investigators group

Lubia Velázquez, Gabriela Ortíz, Andrea Velázquez, María Fernanda Ruiz, María Fernanda Flores, Ninozhka L Rodríguez, Juan P Velasco, Eulalio Rodríguez, Alexa G Paz y Andrea Mauricio. Hospital Regional No 1. “Dr. Carlos MacGregor Sánchez Navarro”. Instituto Mexicano del Seguro Social.

Abril Violeta Muñoz Torres Departamento de Salud Pública. Facultad de Medicina: Universidad Nacional Autónoma de México, (UNAM).

Juana Marlen Ruiz Batalla, Jafet Montero Lara. Clínica de Medicina Familiar No 1. Instituto Mexicano del Seguro Social.

Sandra Vega García, Elizabeth Rivero García, Mariana Villareal Ávalos. Clínica de Medicina Familiar No 7. Instituto Mexicano del Seguro Social.

Diego Arturo Oliva Reyes, Xóchitl Mondragón Sandoval, Maria Guadalupe Suarez Cruz. Clínica de Medicina Familiar No 4. Instituto Mexicano del Seguro Social.

Sergio Luis Garcia Cabazos, Carlos Noé Pino Hernández, Mariana López Delgado. Clínica de Medicina Familiar No 9. Instituto Mexicano del Seguro Social.

Yarenis Santiago Escobar. Edyalit G, Santos Balbuena, Jessica S Aguilar García. Clínica de Medicina Familiar No 28. Instituto Mexicano del Seguro Social.

Julio Antonio Flores Piña, Eloy Olivares Mosco. Emilio Sánchez Huerta. Clínica de Medicina Familiar No.10. Instituto Mexicano del Seguro Social.

## Dietitian working group

Lubia Velázquez López, Gabriela Ortiz Ortíz, María Fernanda Ruiz Mafud, Andrea Velázquez Flores, María Fernanda Flores, Renata Achar de la Macorra.
